# Neurogrit Gold Attenuates 6‐OHDA‐Induced Dopaminergic Neurodegeneration in Parkinson's Model of *Caenorhabditis elegans* by Reducing α‐Synuclein Accumulation and *Pink/Pdr‐1* Driven Mitochondrial Dysfunction

**DOI:** 10.1111/cns.70401

**Published:** 2025-05-08

**Authors:** Acharya Balkrishna, Nishit Pathak, Rani Singh, Vivek Gohel, Yash Varshney, Rishabh Dev, Anurag Varshney

**Affiliations:** ^1^ Drug Discovery and Development Division Patanjali Research Foundation Haridwar India; ^2^ Department of Allied and Applied Sciences University of Patanjali Haridwar India; ^3^ Patanjali Yog Peeth (UK) Trust Glasgow UK; ^4^ Special Centre for Systems Medicine Jawaharlal Nehru University New Delhi India

**Keywords:** 6‐OHDA, autophagy, *Caenorhabditis elegans*, levodopa, mitochondria, Neurogrit gold, Parkinson's disease

## Abstract

**Introduction:**

Parkinson's disease (PD) is a neurodegenerative disorder majorly associated with movement and behavioral disturbances. Pathologically, the loss of dopaminergic (DA) neurons triggered by the deposition of α‐synuclein (SNCA) leads to the decrease in dopamine levels affecting motor and cognitive functions of the brain. Current pharmacotherapy for PD only addresses its symptoms but is not able to halt its progression. Traditional medicines are being increasingly used for the treatment of neurodegenerative disorders.

**Aim:**

The present study investigated the effects of Neurogrit Gold (NG), a herbo‐mineral prescription medicine, on a Parkinson's model of *Caenorhabditis elegans* .

**Methods:**

Chemical characterization of NG was performed on HPLC and GC–MS/MS platforms. Evaluation of NG was done in the neurotoxicant 6‐OHDA‐induced N2, BZ555, and NL5901 strains of *C. elegans* .

**Results:**

It was observed that NG treatment did not hamper the lifespan, survival, and progeny development of *C. elegans* strains. The worms treated with NG were able to resist the deleterious effects of 6‐OHDA on survival, progeny development, body bends, and chemotaxis in N2 and DA neuron degeneration in BZ555 worms. In NL5901 worms, NG treatment reduced SNCA aggregation, restored lipid content, as well as improved body bends, chemotaxis, and food uptake. Gene expression studies on 6‐OHDA exposed and NG‐treated N2 worms suggest that the neuroprotective effects of NG stem from its ability to regulate genes involved in mitochondrial autophagy (*pink‐1*, *pdr‐1*); dopamine synthesis (*cat‐2*); redox (*sod‐3*) and protein folding homeostasis (*hsf‐1*, *hsp‐12.3*).

**Conclusion:**

Neurogrit Gold has robust neuroprotective effects, making it a suitable treatment option against etiologies of Parkinson's disease.

## Graphical abstract

The study describes the therapeutic potential of Neurogrit Gold as a neuroprotective agent in the context of Parkinson's disease (PD). NG reduced α‐synuclein protein aggregation in NL5901 *C. elegans* . Furthermore, NG improved mitochondrial health in 6‐OHDA‐induced dopaminergic neurodegeneration in wild‐type worms and prevented DA neurodegeneration in BZ555 worms.

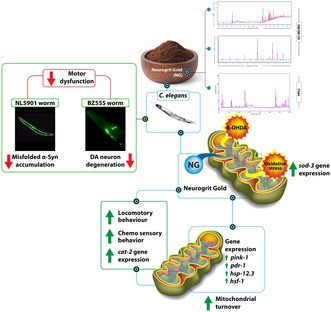

## 1 Introduction

Parkinson's disease (PD) is one of the most prevalent neurodegenerative disorders mostly observed in 2%–3% of the population aged ≥ 65 years. In PD, the loss of dopaminergic (DA) neurons in the substantia nigra pars compacta region of the brain causes striatal dopamine deficiency. Neuronal eosinophilic inclusions known as Lewy bodies and the aggregation of α‐synuclein (SNCA) protein are the neuropathological hallmarks of PD [1, 2, 3]. Clinically, PD is majorly characterized by the presence of rigidity (stiffness of trunk and limbs); bradykinesia (slowness of movement); tremor (trembling in arms, legs, hands, and face); and postural instability (impairment in coordination and balance) [4]. Major molecular pathologies associated with PD are the proteostasis of SNCA, mitochondrial dysfunction, oxidative stress, aberrant autophagy, and loss of nigrostriatal tyrosine hydroxylase (TH) [1, 5, 6]. The current approved pharmacological interventions for PD include the use of levodopa, monoamine oxidase‐B (MAO‐B) inhibitors, and dopamine receptor agonists. However, these interventions only provide symptomatic relief and are not able to halt the progression of the disease [7]. Hence, the development of novel therapeutics that can affect all the key molecular pathologies of PD is required.

The nematode *Caenorhabditis elegans* is used as a model organism to screen anti‐PD drugs as it amalgamates the ease of cell culture with the complexities of multi‐cellular organisms without the ethical obligations related to vertebrate animals. The nematode shares 60%–80% genetic homology with humans as well as structurally and functionally similar DA neuronal pathways, consisting of two anterior and two posterior deirid neurons and four anterior cephalic neurons [8]. Various *C. elegans* models of PD are developed either by exposure to neurotoxicants like 6‐OHDA or MPP^+^ or by use of transgenic strains to mimic the neuropathologies in PD [9]. These worm models exhibit similar phenotypic abnormalities observed in PD patients, like reduced levels of dopamine, degeneration of dopamine neurons, decreased survival, SNCA aggregation, and movement alterations [10, 11, 12, 13]. Transgenic *C. elegans* strains, BZ555 [egIs1 (dat‐1p::GFP)] and NL5901 [pkIs2386 (unc‐54p::alphasynuclein::YFP + unc‐119(+))] are also used to study the localization of DA neurons and SNCA accumulation, respectively while screening for anti‐PD agents [14].

The use of traditional Ayurvedic medicines for the treatment of various ailments has been on the rise [15]. Neurogrit Gold (NG) is an Ayurvedic prescription medicine for the treatment of PD. It is an herbo‐mineral drug comprising extracts of *Tinospora cordifolia* (stem) and *Celastrus paniculatus* (seed) along with classical Ayurvedic preparations, namely *Ekangveer Ras*, *Moti Pishti*, *Rajat Bhasma*, *Vasant Kusumakar Ras*, and *Rasraj Ras* (Table 1). These mineral preparations (*Bhasma*) are treated with herbal decoction or juices and exposed to a certain quantum of heat, as per the classical ancient medicinal texts of Ayurveda. These formulations have been described to treat an array of neurological disorders [16, 17].

**TABLE 1:** Neurogrit Gold (NG) composition.

S.No.	Vernacular name	Scientific name	Book Ref.	Page No.	Qty. (mg)/capsule
1	*Ekangveer Ras*	Classical Preparation	A.F.I.‐I	259	85
2	*Moti Pishti*	Classical Preparation	A.S.S.	145–147	34
3	*Rajat Bhasma*	Classical Preparation	A.F.I.‐I	239–240	34
4	*Vasant Kusumakar Ras*	Classical Preparation	A.F.I.‐I	80–83	17
5	*Rasraj Ras*	Classical Preparation	A.F.I.‐I	270	17
6	Giloy stem Extract	*Tinospora cordifolia*	B.P.N.	258–259	161
7	Jyotishmati Seed Extract	*Celastrus paniculatus*	B.P.N.	86	152

Abbreviations: A.F.I‐I, Ayurvedic Formulary of India‐I, 2nd Edition; A.S.S, Ayurveda Sar Sangrah, Edition‐2010; B.P.N, Bhavprakash Nighantu, Edition‐2010.

The present study investigated the pharmacological effects of NG in 6‐OHDA exposed N2 *C. elegans* , along with BZ555 and NL5901 strains of the nematode. A thorough chemical characterization of NG was performed by HPLC and GC–MS/MS analysis, and subsequently, its pharmacological effects were assessed. The effect of NG was evaluated on the lifespan, adult (%), and progeny development in all the strains of *C. elegans* . Thereafter, an assessment of NG against 6‐OHDA‐induced defects in the developmental, motor, and behavioral patterns of N2 worms was conducted. Additionally, the BZ555 strain was used to analyze the neuroprotective role of NG against 6‐OHDA‐induced damage to DA neurons. The NL5901 strain of *C. elegans* was also employed to evaluate NG effects on SNCA accumulation, lipid deposition, and SNCA‐induced impairments in motor and behavioral patterns. In parallel experiments, levodopa (L‐dopa), a standard‐of‐care PD pharmacotherapy, was used as an experimental control [18] and evaluated with the observed effects of NG.

## 2 Materials and Methods

### 2.1 Reagents

NG (batch #ANGG210005) was sourced from Divya Pharmacy, India. Standards for HPLC and GC–MS/MS analysis: Magnoflorine was purchased from Chem faces, China; β‐Ecdysone was purchased from PHY‐proof, Germany; Palmatine and Piperine were purchased from Sigma‐Aldrich, USA; squalene was purchased from TCI, India. Reagents 5‐fluoro‐2′‐deoxyuridine (FUDR) and 6‐Hydroxydopamine hydrochloride (6‐OHDA) were purchased from Santa Cruz Biotech, USA. Ascorbic acid was purchased from SRL, India. The 3‐(3,4‐Dihdroxyphenyl)‐L‐alanine (levodopa, L‐dopa) was purchased from TCI, India. Nile red was procured from HiMedia, India.

### 2.2 Chemical Analysis of NG on the HPLC Platform

500 mg of powdered NG was transferred to a 10 mL volumetric flask and 7 mL of methanol and water (80:20) was added to it. The solution was sonicated for 30 min, cooled, and diluted with the same diluent. The solution was then centrifuged for 5 min at 5000 rpm and filtered using a 0.45 μm nylon filter. The filtered solution was used for the analysis. Stock solutions (1000 μg/mL each) of Magnoflorine (Potency 98.0% w/w), Palmatine (Potency 75.4% w/w), β‐Ecdysone (Potency 99.9% w/w), and Piperine (Potency 99.8% w/w) were prepared by dissolving accurately weighed standards in methanol. 0.05 mL of each standard was mixed and diluted to 1 mL to prepare a 50 μg/mL concentration of standard mix working solution. The quantification of marker compounds was performed by HPLC (Prominence‐i LC‐2030c 3D Plus, Shimadzu, Japan). The elution was carried out at a flow rate of 1.0 mL/min using gradient elution of mobile phase A (0.1% orthophosphoric acid in water; adjust pH 2.5 by diethylamine) and mobile phase B (Acetonitrile). This experiment was performed on a Shodex C18‐4E (4.6 × 250 mm, 5 μm) column. Gradient programming of the solvent system for mobile phase A was set as 95%–90% for 0 to 10 min, 90%–82% from 10 to 20 min, 82%–65% from 20 to 30 min, 65%–55% from 30 to 50 min, 55%–25% from 50 to 60 min, 25%–10% from 60 to 65 min, 10%–95% from 65 to 66 min, and 95% from 66 to 70 min. 10 μL of standard and test solution were injected, and column temperature was maintained at 35°C. Wavelengths were set at 270 nm for Magnoflorine, Palmatine, and Piperine and 247 nm for β‐Ecdysone.

### 2.3 Chemical Analysis of NG on GC–MS/MS Platform

Analysis was performed on GC–MS/MS (7000D GC/MS triple quad with 7890B GC system) of Agilent‐USA with mass hunter software. Separation was carried out using an HP‐5MS capillary column (30 m × 0.25 mm, 0.25 μm). Helium was used as carrier gas at a flow rate of 1 mL/min. The temperature of the split injector was 280°C, and the split ratio was 20:1. The injection volume was 1 μL. The column temperature was set at 70°C (hold 1 min) and then programmed at 6°C/min to 160°C (hold 3 min), followed by 4°C/min to 300°C (hold 6 min). The GC–MS ion source temperature was 230°C, and the ionization potential was 70 eV. 500 mg of NG was diluted in 5 mL of Ethyl acetate and mixed well. The solution was centrifuged for 5 min at 8000 rpm, filtered through a 0.45 μm nylon filter, and injected. From the 1000 ppm squalene stock solution, a 100 ppm working standard solution in ethyl acetate was prepared.

### 2.4 Chemical Analysis of NG on GC–MS/MS Platform for Fatty Acids

Analysis was performed on GC–MS/MS (7000D GC/MS triple quad with 7890B GC system) of Agilent‐USA with mass hunter software. Separation was carried out using an HP‐88 capillary column (60 m × 0.25 mm, 0.20 μm). Helium was used as carrier gas at a flow rate of 1 mL/min. The temperature of the split injector was 240°C, and the split ratio was 500 mL/min. The injection volume was 1 μL. The column temperature was set at 70°C (hold 3 min) and then programmed at 8°C/min to 230°C (hold 7 min). The GC–MS ion source temperature was 230°C, and the ionization potential was 70 eV. Sample preparation for Fatty Acid Methyl Ester (FAME) as per USP 35 < 401 > Fats and fixed oil. Transferred about 128.3 mg of NG to a 50 mL conical flask fitted with a suitable water‐cooled reflux condenser and a magnetic stir bar. Added 4 mL of 0.5 N Methanolic Sodium Hydroxide solution and refluxed until fat globules disappeared (usually 5–10 min). Added 5 mL of a solution prepared by dissolving 14 g of Boron Trifluoride in methanol to make 100 mL, swirled to mix, and refluxed for 2 min. Added 4 mL of chromatographic n‐heptane through the condenser, and refluxed for 1 min. Cooled, removed the condenser, added 15 mL of saturated Sodium Chloride solution, shook, and allowed the layers to separate. Passed the n‐heptane layer through 0.1 g of Anhydrous Sodium Sulfate (previously washed with chromatographic n‐heptane) into a suitable flask. Transferred 1.0 mL of this solution to a 10 mL volumetric flask, diluted with chromatographic n‐heptane to volume, and mixed. Then transferred 0.05 mL Supelco 37 Component FAME MIX reference standard (Product ID; CRM47885), diluted in 1 mL n‐heptane in GC vial, and injected 1 μL in GC–MS/MS.

### 2.5 Maintenance and Treatment of *Caenorhabditis elegans*

Wild‐type Bristol N2 *C. elegans* , transgenic BZ555 (Pdat‐1::GFP), NL5901 (Punc‐54::‐Syn::YFP) worms, and *E. coli* OP50 were procured from Caenorhabditis Genetic Center (University of Minnesota). *C. elegans* were maintained on NGM plates seeded with *E. coli* OP50 at 20°C [19], in the laboratory incubator.

### 2.6 Lifespan, Adult (%), and Fecundity Assay

NG was dissolved in dimethyl sulfoxide (DMSO), and the final concentration of DMSO was maintained at < 0.3% in all experiments. For life span assay, age‐synchronized L1 (N2, NL5901, and BZ555) worms (*n* = 30) were exposed to different pharmacological doses of NG (1, 3, 10, 30, and 100 μg/mL) on *E. coli* OP50 seeded NGM plate containing 50 μM FUDR (used to prevent progeny production) [20]. After every 5 days, live worms were scored manually by gentle prodding using a metal loop. The life span assay was assessed by the endpoint of the percentage of survived worms with three independent experiments. NG doses 3, 10, and 30 μg/mL were used for further studies.

For adult (%), L1 (N2, NL5901, BZ555) worms were treated with different doses of NG (3, 10, and 30 μg/mL) on an NGM plate seeded with *E. coli* OP50 until they reached the L4 larval stage. The determination of adult (%) was done by counting the number of worms on Day 1 that developed into adult worms on Day 5. Worms were scored manually using the ZEISS Stemi 305 stereo microscope (Carl Zeiss).

In the Fecundity assay (number of progeny), L1 (N2, NL5901, BZ555) worms were treated with different doses of NG (3, 10, and 30 μg/mL) on NGM plates seeded with *E. coli* OP50. After exposure, L4 adult worms were transferred to NGM plates with or without NG and allowed to lay eggs for 24 h. After 24 h, parent worms were removed from the plates, and the progeny were allowed to develop for the next 48 h; thereafter, progeny were counted manually using the ZEISS Stemi 305 stereo microscope (Carl Zeiss). Data were presented as mean ± Standard Error of the Mean (SEM).

### 2.7 Treatment of *C. elegans*

Synchronized N2 and BZ555 worms (L1‐stage) were exposed to different doses of NG (3, 10, and 30 μg/mL) or L‐dopa (2 mM) for 24 h. After incubation, the worms were transferred to an NGM plate seeded with *E. coli* OP50 with or without 50 mM 6‐OHDA and 10 mM ascorbic acid and treated with NG (3, 10, and 30 μg/mL) or L‐dopa (2 mM). After 5 days of exposure, different toxicity parameters (adult % and Fecundity assay) were analyzed. Ascorbic acid (10 mM) was added during 6‐OHDA exposure to minimize its auto‐oxidation. In the case of NL5901 strain of *C. elegans* , treatment with NG (3, 10, and 30 μg/mL) or L‐dopa (2 mM) was performed for 5 days. Unless specified otherwise, this treatment procedure was kept the same for further analysis.

### 2.8 Assessment of DA Neuron Degeneration

After treating the BZ555 worms with 6‐OHDA and NG/L‐dopa, the green fluorescent images were captured by an Olympus BX43 microscope equipped with an FITC filter and a Mantra imaging platform (PerkinElmer) and further processed on the Inform 2.2 software suite (PerkinElmer) and the fluorescence intensity in the head region of 8 worms was quantified using ImageJ software (NIH).

### 2.9 Body Bend Behavior and Chemotaxis

The treated worms were washed with M9 buffer [21] and transferred on NGM plate without food. After a 1 min recovery period, the body bend of individual worms was scored manually for 30 s under a stereo microscope. The experiment was repeated thrice with 10 worms for each group. A chemotaxis assay was performed on a 60 mm NGM plate. 100% ethanol was added to the attractant spot and 1% SDS was added to the repellent spot before the assay. Sodium azide (10 μL of 125 mM) was added 10 min before the assay at the gradient peak to immobilize the worms. After exposure, treated worms were washed with M9 buffer and 30 worms were placed at the center of the NGM plate. Worms were allowed to move over the agar surface for 2 h. After that, worms were scored on each gradient peak and the chemotaxis index (CI) was calculated. Data were presented as mean ± SEM.

### 2.10 Determination of Food Uptake

Food uptake in *C. elegans* was quantified by exposing ~1000 NL5901 L1 worms in a liquid medium (bacteria suspended in M9 medium with or without NG or L‐dopa). The optical density of the suspension was measured at 0 h and 72 h at 600 nm. Data were presented as mean ± SEM.

### 2.11 SNCA Protein Aggregation and Nile Red Staining

SNCA protein aggregation and Nile red staining were assessed in untreated, NG (30 μg/mL) or L‐dopa (2 mM) treated transgenic NL5901 worms. A stock solution of 500 μg/mL Nile Red was prepared in acetone and further diluted 1:500 in the *E. coli* OP50 suspension used as food for worms. After exposure, adult worms (Day 3) were transferred onto the Nile red‐stained *E. coli* OP50‐seeded plates and allowed to grow for 24 h at 20°C in the dark. Thereafter, worms were washed, and images were captured for SNCA aggregation and Nile red by FITC and Texas red filters by Olympus BX43 microscope equipped with a Mantra imaging platform (PerkinElmer) and further processed on Inform 2.2 software suite (PerkinElmer). Images of 10 individual worms were taken at 100 × magnification and quantified using ImageJ software (NIH).

### 2.12 Gene Expression Analysis

Post‐treatment, *C. elegans* were placed in TRIzol. RNA isolation, cDNA synthesis, and real time (RT)‐PCR were performed as previously described [19]. The intensity of fluorescence was captured at each cycle using a Real‐Time System Machine (qTOWER3G, Analytik‐Jena). Primers used for the study are mentioned in Table 2. The *act‐1* gene was used as a housekeeping gene.

**TABLE 2:** Primer sequences for real time PCR.

Gene name	Sequence
*pink‐1* Fd	CAAGGCGAGCCTGAAAGGA
*pink‐1* Rev	GCCGAGAATATTTCCCGCCA
*pdr‐1* Fd	GACTACAAGGTGATCTCAGCGA
*pdr‐1* Rev	CGTGGCATTTTGGGCATCTT
*sod‐3* Fd	AGCATCATGCCACCTACGTGA
*sod‐3* Rev	CACCACCATTGAATTTCAGCG
*cat‐2* Fd	GCCAATGTTCTCGGATCCAC
*cat‐2* Rev	CCGTCGACAGCTTCTCAATG
*hsp‐12.3* Fd	GCCATTCCAGAAAGGAGATG
*hsp‐12.3* Rev	CGTTTGGCAAGAAGTTGTGA
*hsf‐1* Fd	ATGCAGCCAGGATTGTCGAA
*hsf‐1* Rev	GCACGTTTTGAGTTGGGTCC
*act‐1* Fd	ACGACGAGTCCGGCCCATCC
*act‐1* Rev	GAAAGCTGGTGGTGACGATGGTT

### 2.13 Data Analysis

Data were expressed as the mean ± SEM and were derived from at least three independent experiments. Analyses were conducted using GraphPad Prism 8.0 software. The normality of the data was ascertained using the Shapiro–Wilk test and the Kolmogorov–Smirnov test. The Kaplan–Meier method was employed for the lifespan analysis. Statistical significance was evaluated using one‐way ANOVA and the Kruskal‐Wallis test with Dunnett's post hoc analysis.

## 3 Results

### 3.1 Chemical Characterization of NG

**FIGURE 1:**
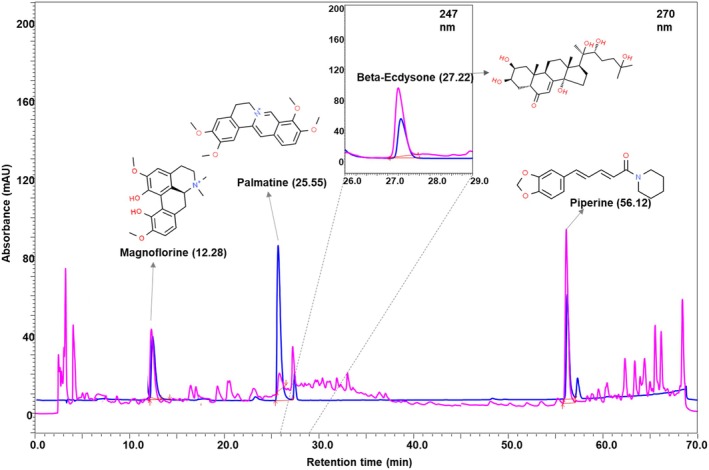
Chemical analysis of Neurogrit Gold (NG) by HPLC. Overlap chromatogram of standard mix (blue line) and NG (pink line) at 247 and 270 nm wavelengths. Quantification of these compounds is mentioned in Table 3.

**FIGURE 2:**
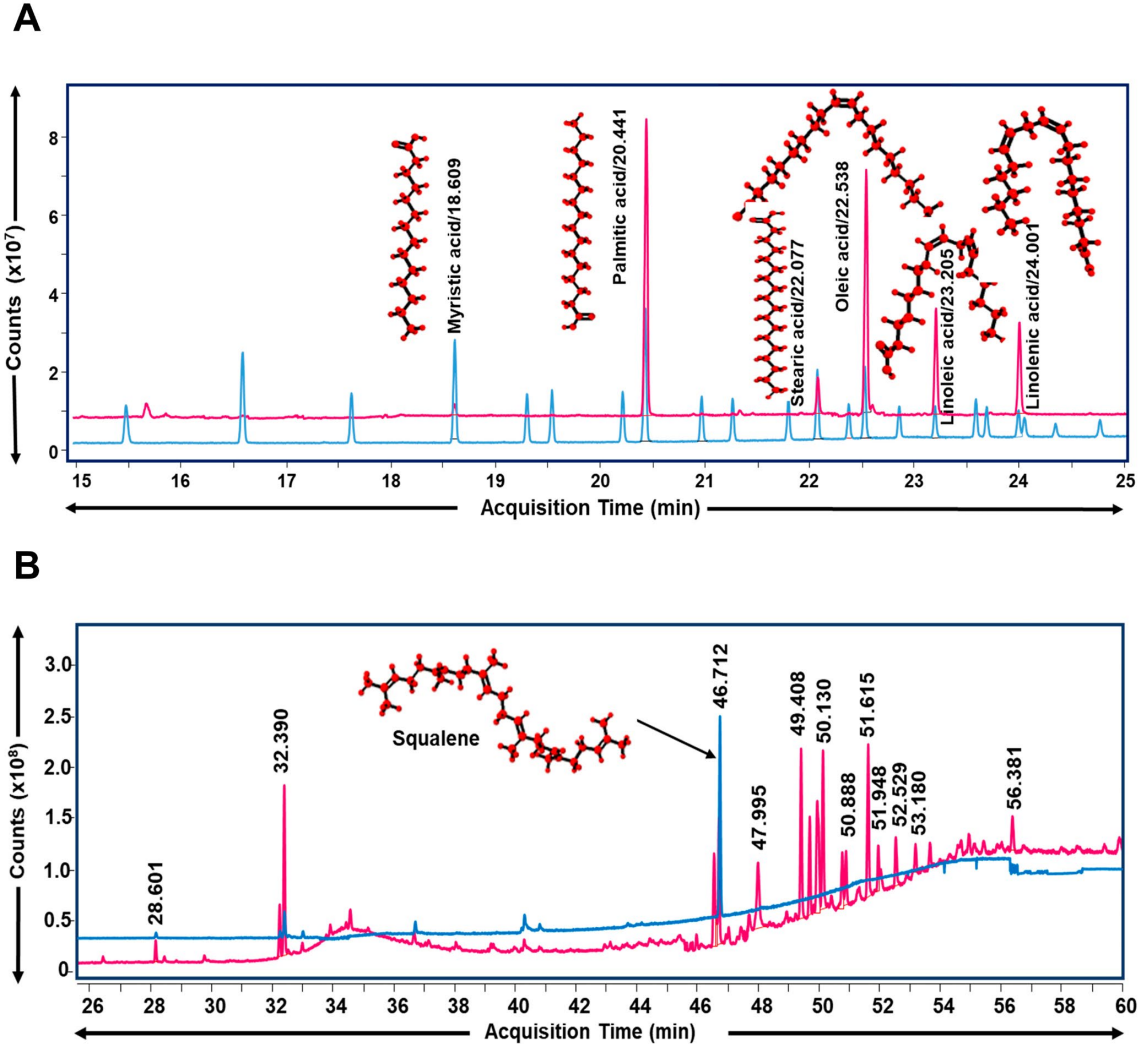
GC–MS/MS‐based chemical characterization of Neurogrit Gold (NG). (A) Overlap chromatogram of the identified fatty acids in NG (pink line) and standard mix (blue line). (B) Chromatogram of the identified compounds in NG (pink line). The identified squalene was further quantified by its standard (blue line). Quantification of these compounds is mentioned in Table 3. Further details of qualitative analysis have been mentioned in Table 4.

The chemical characterization in NG performed using HPLC showed the presence of bioactive compounds as follows: Magnoflorine (RT: 12.28 min), Palmatine (RT: 25.55 min), β‐Ecdysone (RT: 27.22 min), and Piperine (RT: 56.12 min) (Figure 1 and Table 3). Further, the ethyl acetate extract of NG was quantified by GC–MS/MS analysis to identify its fatty acid contents based on their RT and mass‐charge ratio (m/z) (Figure 2A). The GC–MS/MS analysis of the ethyl acetate extract of NG (Figure 2A and Table 3) revealed the presence of fatty acids, viz. Myristic acid (RT: 18.609 min), Palmitic acid (RT: 20.441 min), Stearic acid (RT: 22.077 min), Oleic acid (RT: 22.538 min), Linoleic acid (RT: 23.205 min), and Linolenic acid (RT: 24.001 min). In addition, the n‐heptane extract of NG was also analyzed using GC–MS/MS, and 16 major phytochemicals were identified (Table 4). Squalene (RT: 46.712 min) was found to be one of the major compounds present in NG among these phytochemicals (Figure 2B).

**TABLE 3:** Quantified compounds in Neurogrit Gold by HPLC and GC–MS/MS.

S.No.	Name of Compounds	Retention Time (RT) (min)	Molecular Formula of Compounds	Content in Neurogrit Gold
HPLC				
1	Magnoflorine	12.28	C_20_H_24_NO_4_	1.12
2	Palmatine	25.55	C_21_H_22_NO_4_	0.05
3	β‐Ecdysone	27.22	C_27_H_44_O_7_	1.93
4	Piperine	56.12	C_17_H_19_NO_3_	1.92
GC–MS/MS				
5	Myristic acid	18.609	C_14_H_28_O_2_	0.04
6	Palmitic acid	20.441	C_16_H_32_O_2_	1.19
7	Stearic acid	22.077	C_18_H_36_O_2_	0.18
8	Oleic acid	22.538	C_18_H_34_O_2_	1.26
9	Linoleic acid	23.205	C_18_H_32_O_2_	0.59
10	Linolenic acid	24.001	C_18_H_30_O_2_	0.63
11	Squalene	46.712	C_30_H_50_	0.079

*Note:* Bioactive compounds 1–4 were identified through HPLC (Figure 1) and are expressed in μg/mg, whereas compounds from 5 to 11 were detected through GC– MS/MS (Figure 2A,B) and are expressed in % w/w.

**TABLE 4:** Identified compounds in Neurogrit Gold by GC–MS/MS.

S.No.	Name of Compounds	Retention Time (RT) (min)	Molecular Formula of Compounds	Area %	Score
1	Methyl 14‐methylpentadecanoate	28.16	C_17_H3_4_O_2_	1.22	85.51
2	Linolelaidic acid, methyl ester	32.239	C_19_H_34_O_2_	2.91	89.27
3	Linoleoyl chloride	32.39	C_18_H_31_ClO	9.55	90.69
4	cis‐Z‐.alpha.‐Bisabolene epoxide	46.551	C_15_H_24_O	5.93	66.86
5	Squalene	46.712	C_30_H_50_	7.66	87.57
6	Piperine	47.995	C_17_H_19_NO_3_	7.74	67.65
7	[3,3‐Dimethyl‐2‐(3‐methylbuta‐1,3‐dienyl)cyclohex‐1‐enyl]methanol	49.408	C_14_H_22_O	10.84	67.4
8	1‐Heptatriacotanol	49.696	C_37_H_76_O	5.97	71.34
9	E‐2‐Hexenyl benzoate	49.947	C_13_H_16_O_2_	11.42	68.17
10	2‐Furoic acid, tridec‐2‐ynyl ester	50.13	C_18_H_26_O_3_	10.74	70.57
11	Undec‐10‐ynoic acid, tridec‐2‐yn‐1‐yl ester	50.762	C_24_H_40_O_2_	3.90	72.85
12	5,8,11,14‐Eicosatetraenoic acid, methyl ester, (all‐Z)—	50.888	C_21_H_34_O_2_	3.58	73.58
13	2‐(7‐heptadecynyloxy)tetrahydro‐2H‐Pyran	51.615	C_22_H_40_O_2_	9.80	67.21
14	7,11‐Hexadecadienal	51.948	C_16_H_28_O	2.86	71.69
15	cis‐5,8,11,14,17‐Eicosapentaenoic acid	52.529	C_20_H_30_O_2_	3.45	71.55
16	cis‐8,11,14‐Eicosatrienoic Acid	56.381	C_20_H_34_O_2_	2.40	73.64

*Note:* Bioactive compounds identified in Neurogrit Gold through GC–MS/MS, as deciphered from the chromatogram shown in Figure 2B.

### 3.2 Determination of NG Dose Range for *C. elegans* Treatment by Analyzing Lifespan, Adult (%), and Progeny Development

**FIGURE 3:**
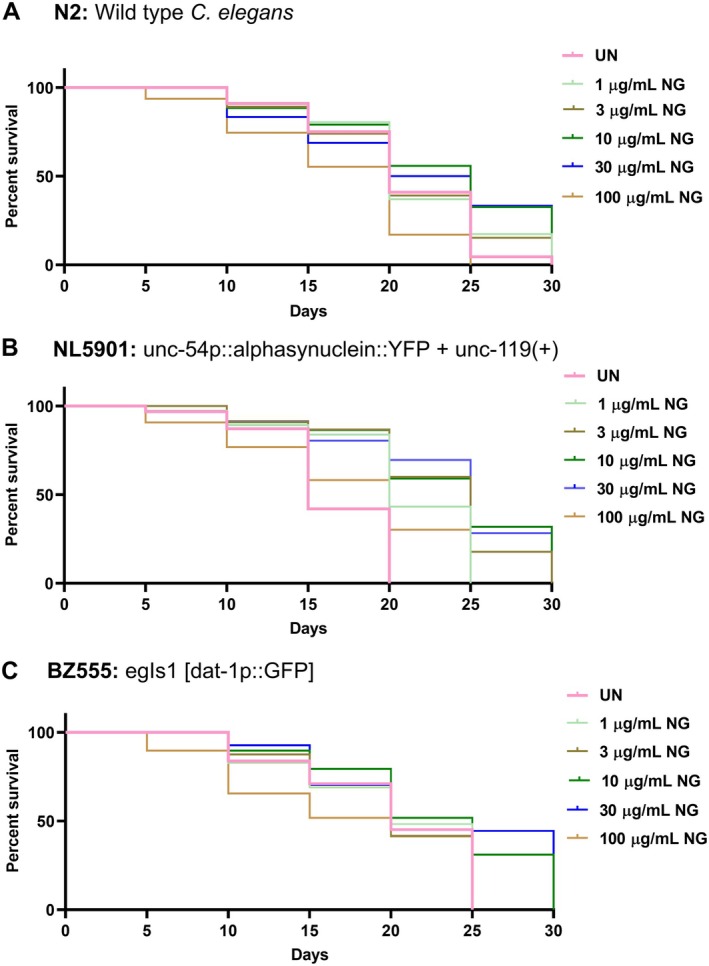
Lifespan analysis. Analysis of lifespan of (A) N2, (B) NL5901, and (C) BZ555 strain of *C. elegans* cultured with different doses of Neurogrit Gold (NG) (1, 3, 10, 30, and 100 μg/mL) as obtained by Kaplan–Meier survival analysis.

**FIGURE 4:**
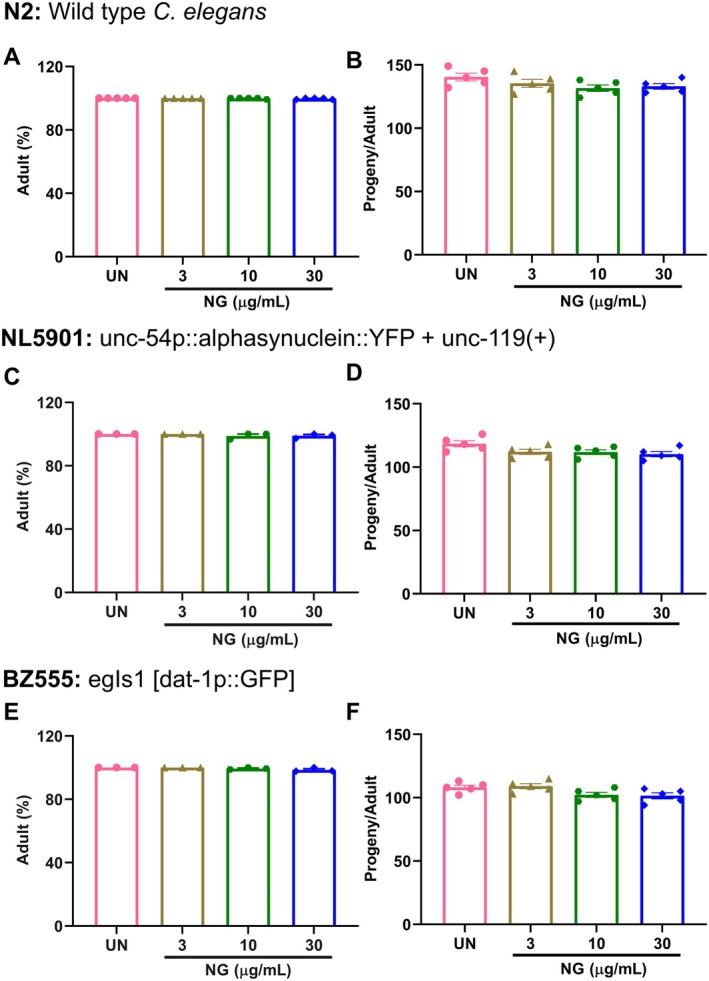
Neurogrit Gold (NG) treatment showed no adverse effect on adult survival and progeny development. Worms were treated with NG (3, 10, and 30 μg/mL) and the adult (%) and progeny development was assessed for (A,B) N2, (C,D) NL5901, and (E,F) BZ555 strain.

Primarily, for the selection of the optimal dose of NG, N2 (wild type), NL5901 (transgenic), and BZ555 (transgenic) strains of *C. elegans* were exposed to different doses of NG (1–100 μg/mL). NG treatment on N2 worms from the L1 larval stage resulted in a decrease in lifespan at the 100 μg/mL dose of NG, whereas no major difference was observed at the other tested doses (1, 3, 10, and 30 μg/mL) compared to untreated worms (Figure 3A). In parallel, an increase in percent survival was observed in all the NG doses (1, 3, 10, 30, and 100 μg/mL) in NL5901 worms (Figure 3B). In BZ555 worms, percent survival at tested doses was either unaltered or found to be enhanced (specifically at 10 and 30 μg/mL) (Figure 3C). Based on lifespan experiments, it was found that NG up to a dose of 30 μg/mL was well‐tolerated for all variants of *C. elegans* used in this study. On that basis, NG doses of 3, 10, and 30 μg/mL were selected for further experimentation. For added validation, the effect of NG (3, 10, and 30 μg/mL) was also evaluated on adult (%) and Progeny development on N2 worms (Figure 4A,B), transgenic NL5901 worms (Figure 4C,D) and BZ555 worms (Figure 4E,F). It was observed that NG at these selected doses had no adverse effects on the lifespan, adult (%), and normal reproduction cycle of *C. elegans* . Therefore, further studies were conducted at these selected doses of NG.

### 3.3 NG Protected N2 Worms From 6‐OHDA Induced Toxicity

**FIGURE 5:**
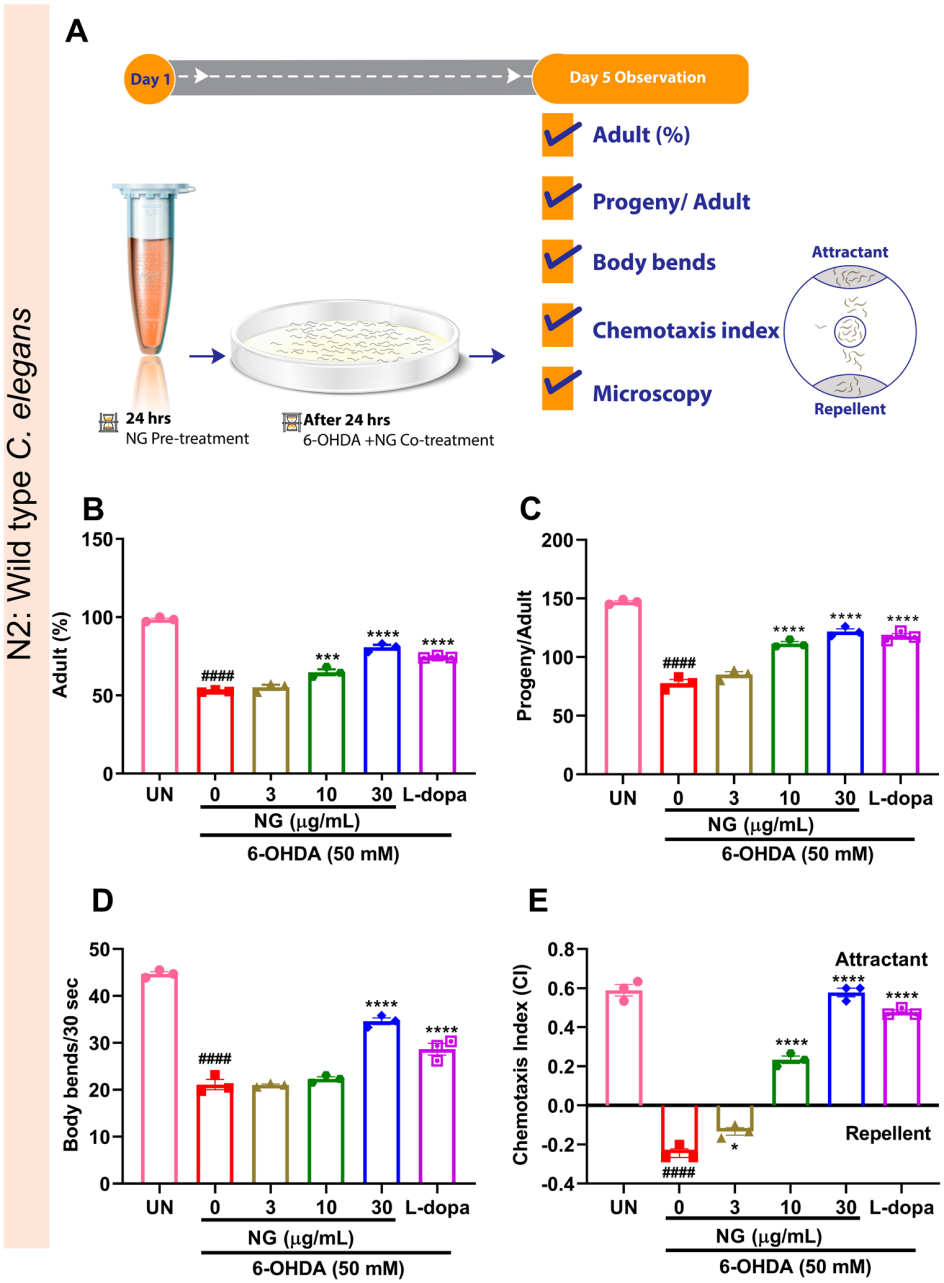
Neurogrit Gold (NG) treated N2 worms resisted the deleterious effects of the neurotoxicant 6‐OHDA. (A) Experimental design and treatment durations. NG (3, 10, and 30 μg/mL) normalized the toxic effects of 6‐OHDA (50 mM) on (B) Adult (%), (C) Progeny development, (D) Body bends, and (E) CI. L‐dopa (2 mM) was used as a positive control. Data represented as mean ± SEM. The significance of data with respect to the untreated (UN) is represented as ####*p* < 0.0001, and with respect to 6‐OHDA (50 mM) is represented as ****p < 0.0001; ***p < 0.001, and *p < 0.05.

6‐OHDA has been well reported in pre‐clinical animal models of PD, as it is known to lower dopamine levels and impair motor functions, the major PD hallmarks [22]. A schematic representation of the effects of NG on various toxicity and behavior parameters of N2 worms exposed to 6‐OHDA has been illustrated in Figure 5A. In the *C. elegans* , stimulation of 6‐OHDA (50 mM) significantly (*p* < 0.0001) reduced adult (%) and progeny development. NG treatment significantly (*p* < 0.0001) enhanced the adult (%) and progeny development at 10 and 30 μg/mL doses of NG in worms exposed to 6‐OHDA (Figure 5B,C). Motor deficits and degeneration of sensory functions (distinctive clinical characteristics of PD) could be analyzed by evaluation of body bends and CI in *C. elegans* [9]. Upon the evaluation of 6‐OHDA‐induced defects in body bends, it was observed that there was a significant (*p* < 0.0001) reduction in the body bends; however, in worms treated with NG exhibited a significant (*p* < 0.0001) protection was exhibited (Figure 5D). DA neuron signaling regulates several behaviors in *C. elegans* , including chemo‐perception, which can be analyzed by evaluation of CI [23]. It was observed that in 6‐OHDA exposed N2 worms, the CI decreased significantly (*p* < 0.001) but upon the addition of NG, it increased significantly (*p* < 0.0001) in a dose‐dependent manner (Figure 5E). Treatment with L‐dopa also showed similar observations (Figure 5B,E). Taken together, NG treatment was able to reduce the toxic neurodegenerative insults of 6‐OHDA in *C. elegans* .

### 3.4 NG Prevented 6‐OHDA Induced Degeneration of Dopaminergic Neurons

**FIGURE 6:**
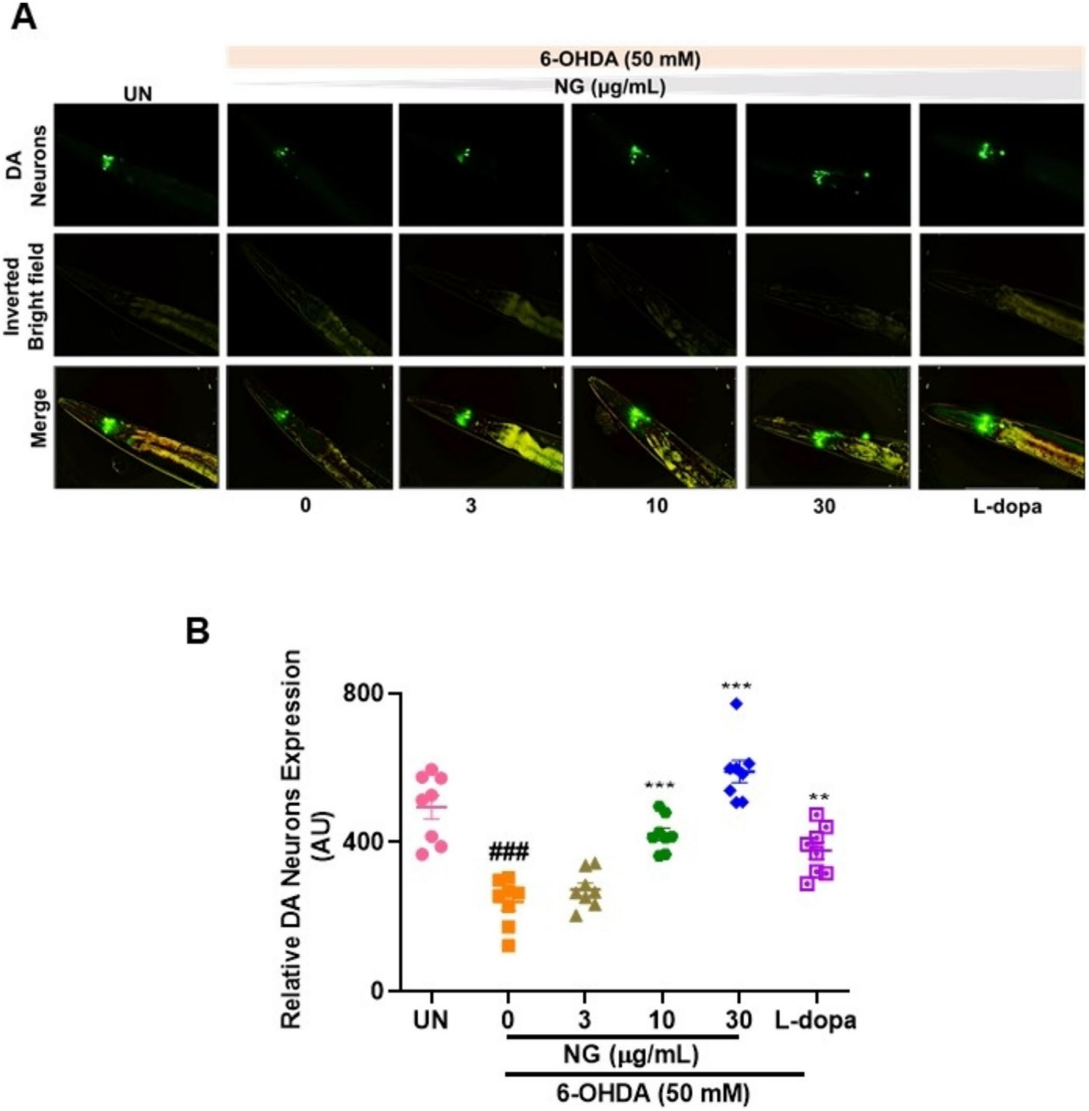
Neurogrit Gold (NG) rescued dopaminergic (DA) neurons from 6‐OHDA‐induced degeneration in transgenic BZ555 strain of *C. elegans* . Post 24 h pre‐treatment with NG (3, 10, and 30 μg/mL) and later 5 days exposure of 50 mM 6‐OHDA along with NG (3, 10, and 30 μg/mL) or L‐dopa (2 mM) the BZ555 strain of worms were analyzed for changes in expression of DA neurons. (A) GFP expression pattern in DA neurons of BZ555. (B) Graphical representation of the fluorescence intensity of DA neurons as quantified by Image J. Data represented as mean ± SEM. The significance of data with respect to the untreated (UN) is represented as ####, *p* < 0.0001 and with respect to 6‐OHDA (50 mM) is represented as ****, *p* < 0.0001, and **, *p* < 0.01.

The neurotoxicant 6‐OHDA selectively induces the degeneration of DA neurons [22, 24, 25]. The damage in DA neurons was observed using the GFP‐tagged transgenic strain BZ555. Untreated worms showed bright GFP fluorescence in the cell bodies and dendrites from the nerve ring to the anterior region of the pharynx. The exposure of 50 mM 6‐OHDA in worms showed damage in the DA neurons, with significantly (*p* < 0.0001) reduced relative mean fluorescence intensity (Figure 6A,B). Treatment with NG also significantly (*p* < 0.0001) elevated the resistance of DA neurons to 6‐OHDA at 10 and 30 μg/mL, as observed from the increase in GFP intensity. L‐dopa‐treated worms showed similar results (Figure 6A,B). Taken together, NG treatment reduced the 6‐OHDA‐induced degeneration of DA neurons.

### 3.5 NG Prevented Accumulation of α‐Synuclein (SNCA) and Increased Lipid Deposition in NL5901 Strain of *C. elegans*

**FIGURE 7:**
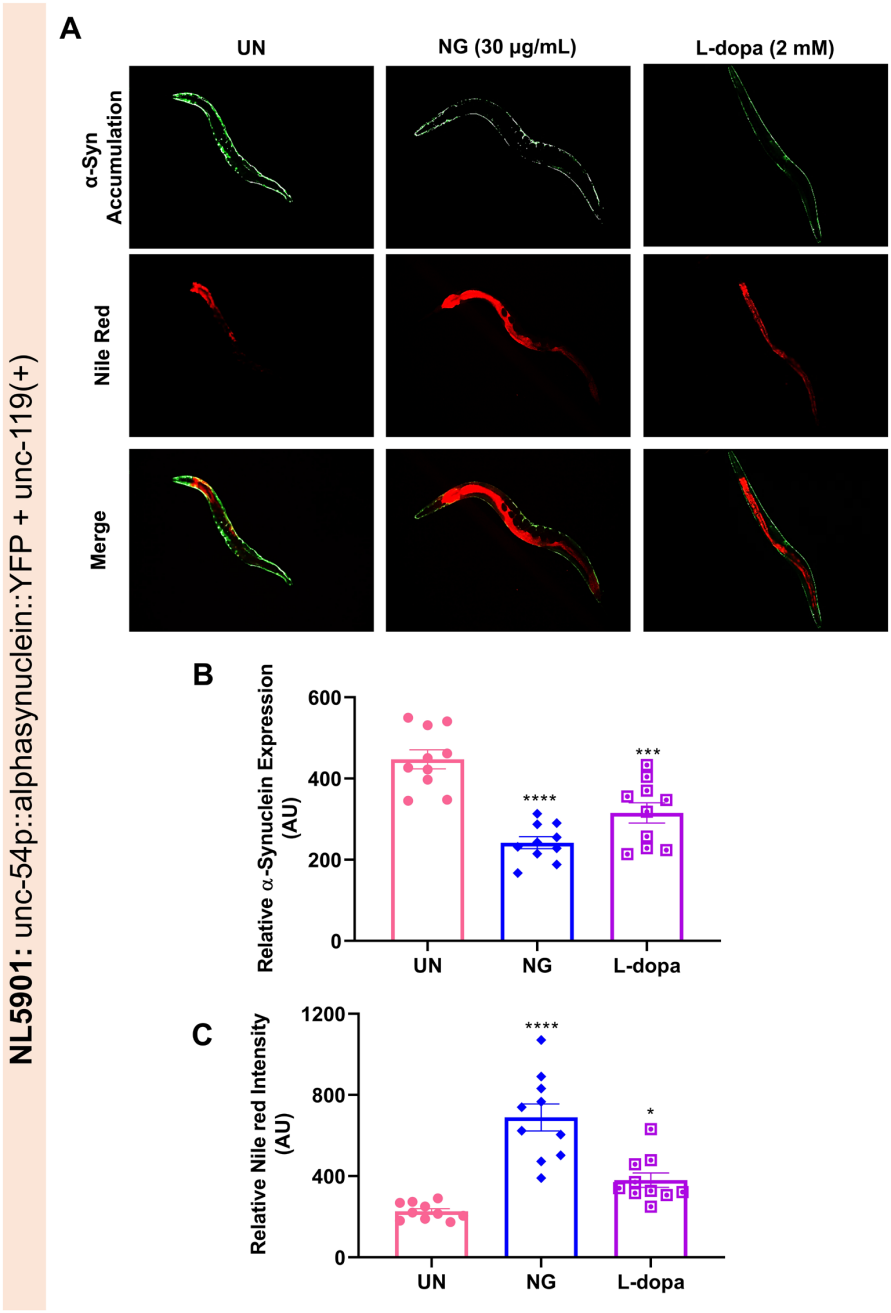
Neurogrit Gold (NG) arrested α‐synuclein (SNCA) aggregation and increased lipid deposition in NL5901 strain of *C. elegans* . (A) SNCA accumulation (GFP) and Nile red based evaluation of lipid deposition in NG (30 μg/mL) or L‐dopa (2 mM) treated NL5901 worms. Quantification of the (B) SNCA and (C) Nile red fluorescence intensity as observed by Image J. Data represented as mean ± SEM. The significance of data with respect to the untreated (UN) is represented as ****p < 0.0001; ***p < 0.001, and *p < 0.05.

In order to evaluate NG against SNCA accumulation, the NL5901 strain of *C. elegans* was utilized. Lewy bodies are mostly composed of aggregated SNCA, and the growth of Lewy bodies in the brain is one of the distinguishing features of Parkinson's disease patients [26]. A remarkable reduction of SNCA accumulation in the body wall muscles of NL5901 worms was observed with NG (30 μg/mL) treatment (Figure 7A). NL5901 worms treated with NG (30 μg/mL) exhibited a significantly (*p* < 0.0001) lower SNCA fluorescence intensity compared to untreated NL5901 worms (Figure 7B). Lipid peroxidation in response to SNCA accumulation is responsible for the decline in lipid moieties required for neurotransmission in the CNS [27, 28]. Upon evaluation of lipid levels in SNCA‐expressing worms and those treated with NG, it was observed that lipid levels inside the NG‐treated worms were visibly higher compared to untreated ones (Figure 7A). The NL5901 worms treated with NG or L‐dopa displayed a significant (*p* < 0.0001 or *p* < 0.05) increase in Nile red fluorescence intensity (Figure 7C). Hence, it was observed that the SNCA‐related pathologies of PD could be mitigated by NG treatment.

### 3.6 NG Improved Dopamine‐Dependent Behavior in NL5901 Strain of *C. elegans*

**FIGURE 8:**
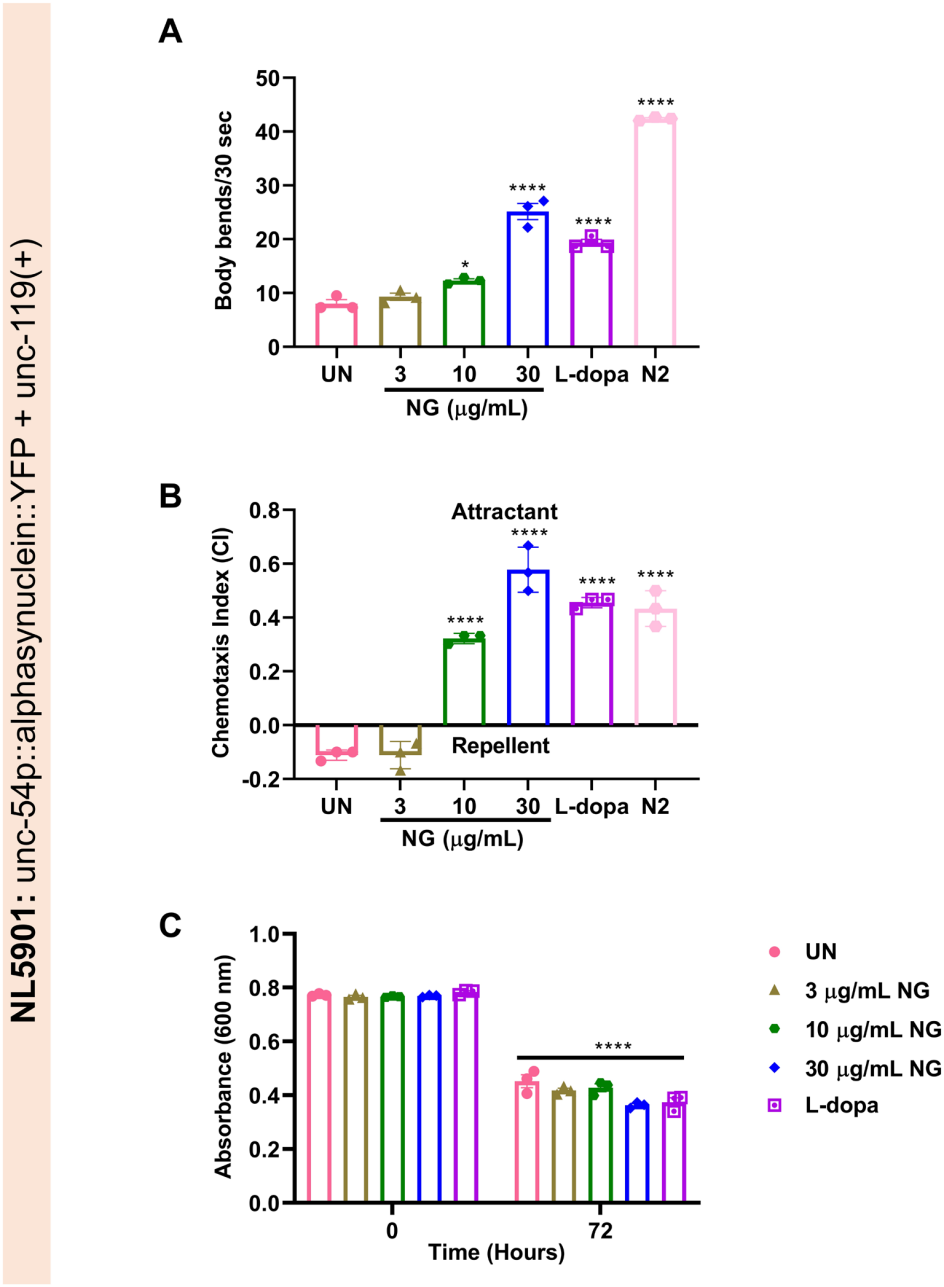
Neurogrit Gold (NG) treatment enhanced mobility, chemosensory, and food uptake behavior of NL5901 strain of *C. elegans* . NG (3, 10, and 30 μg/mL) or L‐dopa (2 mM) treatment enhanced (A) Body bends, (B) Chemotaxis index, and (C) Food uptake parameters of NL5901 worms. Data represented as mean ± SEM. The significance of data with respect to the untreated (UN) is represented as ****p < 0.0001, and *p < 0.05.

Mutant SNCA transgenic *C. elegans* display a behavioral phenotype of decreased mobility, food‐sensing behavior, and chemotaxis. This behavior is a dopamine‐specific adaptive response to food intake by the worms [29, 30, 31]. As observed before in 6‐OHDA‐induced N2 worms, the motor and chemosensory parameters, namely body bends and CI, also showed a significant (*p* < 0.0001) increase upon treatment with NG in NL5901 worms (Figure 8A,B). Furthermore, as deficits were observed in the chemosensory behavior of the NL5901 worms, the amount of their food uptake was also observed. Initially, no significant change was observed in the levels of food uptake at 0 h. So, the baseline was similar for all the treatment groups. Next, it was observed that after 72 h treatment of NG (30 μg/mL) or L‐dopa, the food uptake was significantly (*p* < 0.0001) enhanced, as found from the reduction in optical density of bacteria left in the plate (Figure 8C). Taken together, NG can ameliorate the characteristics of PD by enhancement of mobility and chemosensory behavior.

### 3.7 NG Enhanced Expression of Genes Involved in the Maintenance of Mitochondrial Health and Redox Homeostasis

**FIGURE 9:**
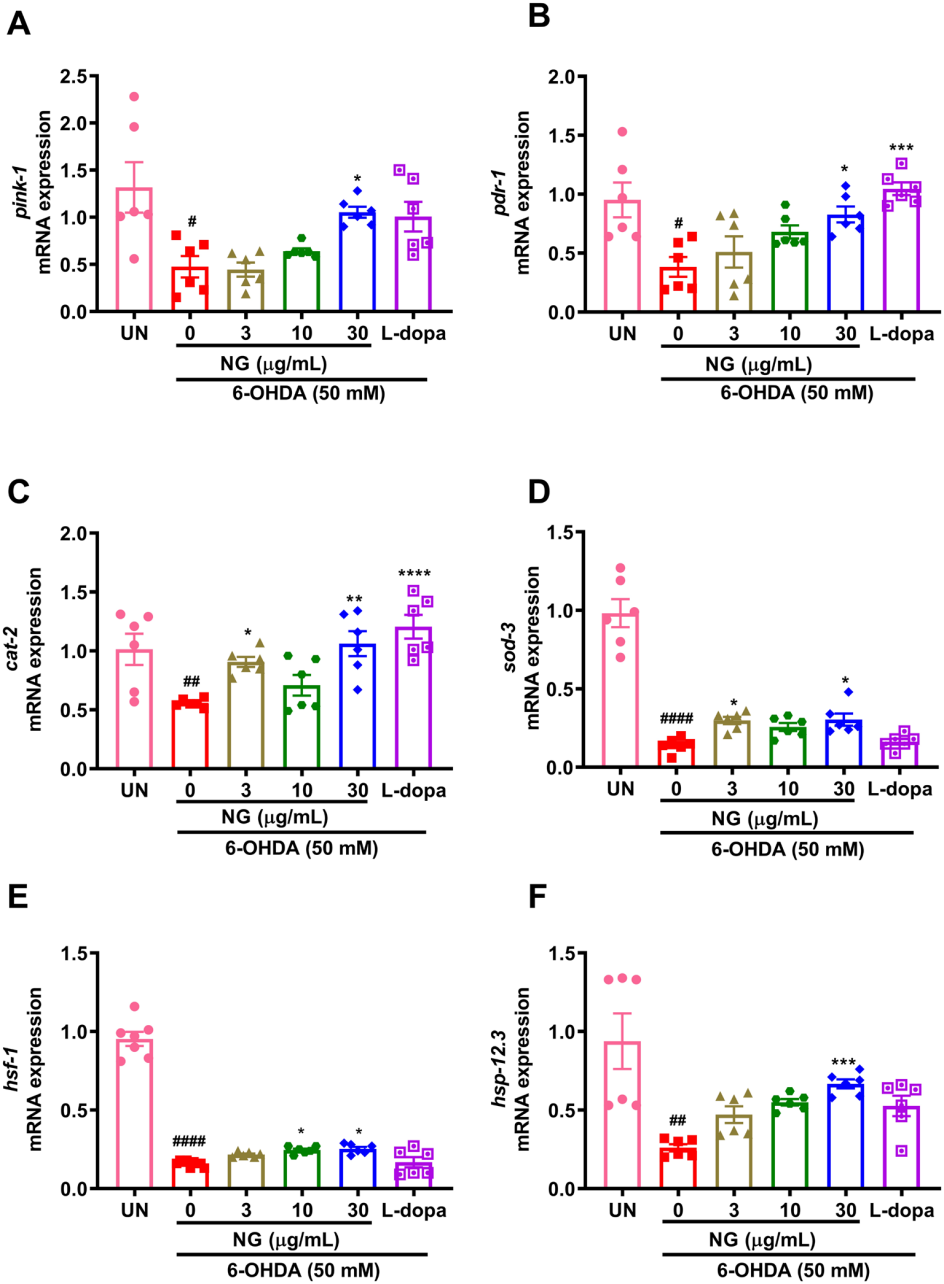
Neurogrit Gold (NG) treatment modulated mRNA expression in 6‐OHDA‐exposed N2 strain of *C. elegans* . Post 24 h pre‐treatment with NG (3, 10, and 30 μg/mL) and later 5 days exposure of 50 mM 6‐OHDA along with NG (3, 10, and 30 μg/mL) or L‐dopa (2 mM), the mRNA expression of N2 worms was analyzed. Gene expression of (A) *pink‐1*, (B) *pdr‐1*, (C) *cat‐2*, (D) *sod‐3*, (E) *hsf‐1*, and (F) *hsp‐12.3*. Data represented as mean ± SEM. The significance of data with respect to the untreated (UN) is represented as ####*p* < 0.0001; ##*p* < 0.01 and #*p* < 0.05 and with respect to 6‐OHDA (50 mM) is represented as ****p < 0.0001; ***p < 0.001; **p < 0.01 and *p < 0.05.

The expression of *pink‐1* and *pdr‐1*, genes responsible for the autophagy of defective mitochondria [32], was significantly (*p* < 0.05) downregulated in 6‐OHDA‐exposed nematodes. In the presence of NG (30 μg/mL) the gene expression significantly (*p* < 0.05) increased (Figure 9A,B). The *cat‐2* gene, which encodes for tyrosine hydroxylase involved in the synthesis of dopamine [33] was found to be significantly (*p* < 0.001) downregulated upon 6‐OHDA exposure to nematodes. In the NG‐treated group, its expression (NG 30 μg/mL; *p* < 0.001) was increased (Figure 9C). Redox imbalance in *C. elegans* is majorly absolved by superoxide dismutase‐3 (*sod‐3*) [19]. The *sod‐3* gene expression was significantly (NG 30 μg/mL; *p* < 0.05) upregulated post‐treatment with NG (Figure 9D). The expression of *hsf‐1* (*p* < 0.0001) and *hsp‐12.3* (*p* < 0.01), genes involved in the management of proteotoxic stress [14], were significantly decreased upon 6‐OHDA exposure, but treatment with NG significantly increased the expression of *hsf‐1* (NG 10 and 30 μg/mL; *p* < 0.05) and *hsp‐12.3* (NG 30 μg/mL; *p* < 0.001) (Figure 9E,F). Thus, NG treatment might be able to resist the neurodegenerative changes due to the loss of DA neurons.

## 4 Discussion

Parkinson's disease (PD) is the second most common neurodegenerative disorder [34]. The current pharmacotherapies for PD majorly provide only symptomatic relief and have been associated with major adverse effects like impulsive and compulsive behavior, dyskinesia, hallucinations, and motor complications [7, 35]. Medicines from ethnopharmacological origins are being increasingly utilized to treat PD due to their safety and multifaceted bioactivities [34, 36]. The current study investigated the anti‐Parkinsonism activity of the herbo‐mineral Ayurvedic prescription medicine NG. The chemical characterization of NG revealed the presence of bioactives like magnoflorine, palmatine, β‐ecdysone, piperine, myristic acid, palmitic acid, stearic acid, oleic acid, linoleic acid, linolenic acid, and squalene. These compounds are known to be effective against molecular etiologies of PD [37, 38, 39, 40, 41, 42]. To further characterize the pharmacological properties of NG, it was evaluated in vivo on various strains of *C. elegans* . Prior to the evaluation of the bioactivity of NG, its effect on the survival, adult (%), and progeny development was assessed on the N2, NL5901, and BZ555 strains of *C. elegans* . This was done to rule out any confounding bias in the obtained results. It was observed that at the selected doses, NG treatment did not alter the normal lifespan, growth, and reproduction parameters of the *C. elegans* .

PD‐like pathologies can be induced in *C. elegans* by the neurotoxin 6‐OHDA, which selectively abolishes DA neurons by inducing oxidative stress and mitochondrial damage [32, 34, 43]. The *C. elegans* (N2) exposed to 6‐OHDA showed a decline in adult (%) and egg‐laying capacity, which were improved by NG treatment in a dose‐dependent manner. Progressive loss of muscle mass and function (Sarcopenia) is observed in older adults with PD. In *C. elegans* , sarcopenia corresponds to reduced mobility that can be assessed by counting the number of body bends per unit time [10]. When *C. elegans* were exposed to 6‐OHDA, a reduction occurred in their number of body bends, whereas in the worms co‐exposed with NG, the number of body bends got normalized. This might be due to the presence of *Tinospora cordifolia* in NG, which is known to restore behavioral changes in locomotion in rats induced with 6‐OHDA [44]. Nearly 90% of PD patients exhibit symptoms of olfactory bulb dysfunction due to the loss of DA neurons. In *C. elegans* , this can be observed by the measurement of attractive or repulsive chemotaxis, which is dependent upon functional chemotaxis [23, 45]. It was observed that worms exposed to 6‐OHDA had a negative CI, i.e., they were not able to differentiate between attractants and repellents; however, worms treated with NG showed a dose‐dependent increase in CI. Palmitic acid, a major constituent in NG, was also observed to alleviate neurotoxicity induced by 6‐OHDA in *C. elegans* [46]. As the neurotoxin 6‐OHDA explicitly degrades the nigral dopamine neurons [47], the neuroprotective efficiency of NG was also analyzed in the BZ555 worms. It was observed that upon exposure to 6‐OHDA in BZ555 worms, the GFP intensity of their DA neurons decreased; however, in the presence of NG, the intensity increased and was normalized at higher doses. This effect of NG can be in response to the presence of Piperine as one of its components. Piperine is reported to protect against 6‐OHDA‐induced PD in a rat model [48]. Additionally, as NG contains gold nanoparticles (*Swarna Bhasma*), its pharmacological effects will be further bolstered due to better penetration through the blood–brain barrier [49, 50].

Lipid moieties in the central nervous system are vital for neurotransmission. Although most energy consumed by brain cells comes from glucose, lipids provide nearly 20% of the total energy consumption of the adult brain. Lipids are associated with several PD etiologies, like oxidative stress, endoplasmic reticulum stress, and endosomal‐lysosomal defects. Changes in lipid composition or content vastly modify the key processes involved in the maintenance of normal neuronal functions [51]. The temporal and spatial aspects of cellular signaling can be modulated by lipid components that can change protein location and scaffolding events by the dynamic control of membrane microdomains. Disarrayed cellular signaling is majorly linked with almost every neurodegenerative disease [52]. It has also been reported that alterations in the lipid specificity of SNCA cause disruptions in the complex network of synaptic machinery. SNCA oligomers induce lipid peroxidation, which disrupts normal cell signaling. Nile red, a lipophilic dye, stains lysosome‐lipid compartments in the intestine, which allows for the study of lysosomal degradation of SNCA in *C. elegans* [27, 28]. The NL5901 strain of the nematode permits direct monitoring of SNCA aggregation throughout the lifespan of the worm as it mimics the SNCA aggregation found in Lewy bodies [10]. It was observed that NG decreased the SNCA accumulation and also increased the Nile red intensity. This might be due to the presence of *Celastrus paniculatus*, a component of NG that is reported to reduce SNCA accumulation and lipid peroxidation in various pre‐clinical studies [53, 54]. To further validate the anti‐PD bioactivities of NG, the defects in motor and sensory‐related pathways were also evaluated in NL5901 by assessment of body bends and CI. It was observed that NG treatment enhanced the body bends and chemotaxis parameters in a dose‐dependent manner. This can be correlated to the presence of *Rajat Bhasma* in NG, which is traditionally used for the treatment of Parkinsonism [55]. In *C. elegans* , *pink‐1* (ortholog of human PINK1) is responsible for the maintenance of the morphology, function, and quality of mitochondria. PINK1 contains a mitochondrial targeting sequence that, under normal conditions, facilitates the translocation of PINK1 to mitochondria, where it senses reactive oxygen species and initiates mitophagy [56]. It is also related to the anti‐apoptotic activity of dopaminergic neurons. *Pdr‐1* (ortholog of human Parkin) found in *C. elegans* is known to be inactivated in the brains of PD patients. It is responsible for the elimination of dysfunctional mitochondria by enhancing autophagy [32]. PDR‐1/parkin acts downstream of PINK‐1 and functions by ubiquitylating outer mitochondrial membrane proteins for their recognition by mitophagy receptors [57]. Aging induces defects in PINK‐1 dependent mitophagy by upregulation of Miro, an outer mitochondrial membrane protein. Miro increases mitochondrial mobility, which compromises mitophagy initiation [58].

Mitochondrial dysfunction and impairment in mitophagy are the cardinal pathological hallmarks of PD [58, 59]. It is reported that the expression of PINK1 and Parkin is reduced in 6‐OHDA‐induced DA neurons [60]. In the current study, it was also observed that the expression of *pink‐1* and *pdr‐1* in 6‐OHDA‐exposed *C. elegans* decreased, but in NG‐treated worms, it increased dose‐dependently. This effect by NG can be correlated to the presence of herbo‐mineral nanoparticles (*Bhasma*) of copper, iron, mica, silver, and gold in its formulation, which are known to promote autophagy [16, 17, 61, 62]. Another gene directly related to the PD‐like pathology in *C. elegans* is *cat‐2*, which encodes tyrosine hydroxylase, a rate‐limiting enzyme for dopamine synthesis [33]. It was observed that upon 6‐OHDA exposure, *cat‐2* expression decreased in the nematodes. However, in the presence of NG, the worms showed a normalized expression of *cat‐2*. Exposure to 6‐OHDA causes mitochondrial failure by inhibition of complex I of the mitochondrial electron transport chain, which results in elevated oxidative stress and ultimately damage to DA neurons [33]. The treatment with NG decreased the 6‐OHDA‐induced alterations in the levels of *sod‐3*, *hsp‐12.3*, and *hsf‐1*, which are involved in the reduction of oxidative stress, prevention of SNCA misfolding, and degradation of SNCA, respectively [14]. This bioactivity of NG plays an essential role in halting the progression of PD. These effects of NG might be due to the presence of *Moti pishti*, which contains mineral pearl powder, known to reduce oxidative stress and target proteins involved in pathways of PD [63, 64].

Despite these data describing anti‐PD properties of NG in *C. elegans‐based* PD models, some limitations should be considered while interpreting the findings of this study. It should be acknowledged that *C. elegans‐based* models represent some facets of PD and do not depict the multifaceted pathophysiology of PD as observed in humans. In addition, the anti‐PD properties of NG observed in this study could well be investigated in mammalian models, with diverse endpoints.

In summary, NG was observed to have bioactivity against PD as assessed in various strains of *C. elegans* . Most of the pharmacological effects of NG were at par with those of levodopa, which is considered to be the standard pharmacotherapy for PD. However, the side effects of levodopa, like dyskinesia, peripheral neuropathy, cognitive decline, and osteoporosis, limit its long‐term use [65]. Taken together, NG exhibited robust multifaceted effects against phenotypes associated with Parkinsonian etiologies. Collectively, these outcomes could pave the way for the in‐depth clinical assessments of NG in patients with Parkinson's disease.

## Author Contributions

**Acharya Balkrishna:** conceptualization, Planning, Visualization, Supervision, Writing – review and editing. **Nishit Pathak:** methodology, Investigation, Formal analysis, Writing – original draft, Writing – review and editing. **Rani Singh:** methodology, Investigation, Formal analysis. **Vivek Gohel:** visualization, Writing – original draft. **Yash Varshney:** methodology, Investigation, Formal analysis. **Rishabh Dev:** data curation, Writing – review and editing, Visualization, Project administration, Supervision. **Anurag Varshney:** writing – review and editing, Project administration, Conceptualization, Visualization, Supervision.

## Ethics Statement

The authors have nothing to report.

## Consent

The authors have nothing to report.

## Conflicts of Interest

The test article (NG) was sourced from Divya Pharmacy, Haridwar, India. Acharya Balkrishna is an honorary trustee in Divya Yog Mandir Trust, which governs Divya Pharmacy, Haridwar. In addition, he holds an honorary managerial position in Patanjali Ayurved Ltd., Haridwar, India. Divya Pharmacy, Haridwar, India, and Patanjali Ayurved Ltd., Haridwar, India, manufacture and sell herbal medicinal products. Other than providing the test formulation (NG), Divya Pharmacy was not involved in any aspect of the research reported in this study. All other authors have declared no conflicts of interest.

## Data Availability

The raw data supporting the conclusions of this article will be made available by the authors, without undue reservation.

## References

[1] W. Poewe , K. Seppi , C. M. Tanner , et al., “Parkinson disease,” Nature Reviews Disease Primers 3, no. 1 (2017): 17013, 10.1038/nrdp.2017.13. 28332488

[2] A. Elbaz , L. Carcaillon , S. Kab , and F. Moisan , “Epidemiology of Parkinson's Disease,” Revue Neurologique (Paris) 172, no. 1 (2016): 14–26. 10.1016/j.neurol.2015.09.012 26718594

[3] Z. Ou , J. Pan , S. Tang , et al., “Global Trends in the Incidence, Prevalence, and Years Lived With Disability of Parkinson's Disease in 204 Countries/Territories From 1990 to 2019,” Frontiers in Public Health 9 (2021): 776847. 34950630 10.3389/fpubh.2021.776847 PMC8688697

[4] F. C. Church , “Treatment Options for Motor and Non‐Motor Symptoms of Parkinson's Disease,” Biomolecules 11, no. 4 (2021): 612. 33924103 10.3390/biom11040612 PMC8074325

[5] A. K. Mishra , M. S. Ur Rasheed , S. Shukla , M. K. Tripathi , A. Dixit , and M. P. Singh , “Aberrant Autophagy and Parkinsonism: Does Correction Rescue From Disease Progression?,” Molecular Neurobiology 51, no. 3 (2015): 893–908. 24833602 10.1007/s12035-014-8744-3

[6] I. Kawahata and K. Fukunaga , “Degradation of Tyrosine Hydroxylase by the Ubiquitin‐Proteasome System in the Pathogenesis of Parkinson's Disease and Dopa‐Responsive Dystonia,” International Journal of Molecular Sciences 21, no. 11 (2020): 3779. 32471089 10.3390/ijms21113779 PMC7312529

[7] E. Angelopoulou , E. S. Pyrgelis , and C. Piperi , “Neuroprotective Potential of Chrysin in Parkinson's Disease: Molecular Mechanisms and Clinical Implications,” Neurochemistry International 132 (2020): 104612. 31785348 10.1016/j.neuint.2019.104612

[8] L. P. D. da Silva , E. da Cruz Guedes , I. C. O. Fernandes , L. A. L. Pedroza , G. J. da Silva Pereira , and P. Gubert , “Exploring *Caenorhabditis Elegans* as Parkinson's Disease Model: Neurotoxins and Genetic Implications,” b 42, no. 1 (2024): 11, 10.1007/s12640-024-00686-3. 38319410

[9] J. F. Cooper and J. M. Van Raamsdonk , “Modeling Parkinson's Disease in *C. elegans* ,” Journal of Parkinson's Disease 8, no. 1 (2018): 17–32. 10.3233/JPD-171258 PMC5836411 29480229

[10] S. Hughes , M. van Dop , N. Kolsters , D. van de Klashorst , A. Pogosova , and A. M. Rijs , “Using a *Caenorhabditis elegans* Parkinson's Disease Model to Assess Disease Progression and Therapy Efficiency,” Pharmaceuticals (Basel) 15, no. 5 (2022): 512. 35631338 10.3390/ph15050512 PMC9143865

[11] P. Chauhan , K. Wadhwa , and G. Singh , “ *Caenorhabditis Elegans* as a Model System to Evaluate Neuroprotective Potential of Nano Formulations,” Frontiers in Nanotechnology 4 (2022): 1018754.

[12] R. Vozdek , P. P. Pramstaller , and A. A. Hicks , “Functional Screening of Parkinson's Disease Susceptibility Genes to Identify Novel Modulators of Alpha‐Synuclein Neurotoxicity in *Caenorhabditis Elegans* ,” Frontiers in Aging Neuroscience 14 (2022): 806000. 35572147 10.3389/fnagi.2022.806000 PMC9093606

[13] J. F. Cooper , D. J. Dues , K. K. Spielbauer , E. Machiela , M. M. Senchuk , and J. M. Van Raamsdonk , “Delaying Aging Is Neuroprotective in Parkinson's Disease: A Genetic Analysis in *C. elegans* Models,” NPJ Parkinsons Disease 1, no. 1 (2015): 15022, 10.1038/npjparkd.2015.22. PMC5516561 28725688

[14] P. Chalorak , T. Sanguanphun , T. Limboonreung , and K. Meemon , “Neurorescue Effects of Frondoside A and Ginsenoside Rg3 in *C. elegans* Model of Parkinson's Disease,” Molecules (Basel, Switzerland) 26, no. 16 (2021): 4843. 34443430 10.3390/molecules26164843 PMC8402114

[15] A. Balkrishna , Y. Rustagi , K. Bhattacharya , and A. Varshney , “Application of Zebrafish Model in the Suppression of Drug‐Induced Cardiac Hypertrophy by Traditional Indian Medicine Yogendra Ras,” Biomolecules 10, no. 4 (2020): 600. 32295034 10.3390/biom10040600 PMC7226110

[16] D. Pal , C. K. Sahu , and A. Haldar , “Bhasma: The Ancient Indian Nanomedicine,” Journal of Advanced Pharmaceutical Technology & Research 5, no. 1 (2014): 4–12. 24696811 10.4103/2231-4040.126980 PMC3960793

[17] S. Farooq , Z. Mehmood , F. A. Qais , M. S. Khan , and I. Ahmad , “Nanoparticles in Ayurvedic Medicine,” in New Look to Phytomedicine, ed. M. S. Ahmad Khan , I. Ahmad , and D. Chattopadhyay (Academic Press, 2019), 581–596.

[18] H. Bogetofte , A. Alamyar , M. Blaabjerg , and M. Meyer , “Levodopa Therapy for Parkinson's Disease: History, Current Status and Perspectives,” CNS & Neurological Disorders Drug Targets 19, no. 8 (2020): 572–583. 32703142 10.2174/1871527319666200722153156

[19] A. Balkrishna , V. Gohel , N. Pathak , et al., “Anti‐Oxidant Response of Lipidom Modulates Lipid Metabolism in Caenorhabditis Elegans and in OxLDL‐Induced Human Macrophages by Tuning Inflammatory Mediators,” Biomedicine & Pharmacotherapy 160 (2023): 114309. 36709598 10.1016/j.biopha.2023.114309

[20] N. Feldman , L. Kosolapov , and A. Ben‐Zvi , “Fluorodeoxyuridine Improves *Caenorhabditis elegans* Proteostasis Independent of Reproduction Onset,” PLoS One 9, no. 1 (2014): e85964. 24465816 10.1371/journal.pone.0085964 PMC3897603

[21] F. He , “Common Worm Media and Buffers,” Bio‐Protocol 1, no. 7 (2011): e55.

[22] A. Schober , “Classic Toxin‐Induced Animal Models of Parkinson's Disease: 6‐OHDA and MPTP,” Cell and Tissue Research 318, no. 1 (2004): 215–224. 15503155 10.1007/s00441-004-0938-y

[23] J. Yan , Z. Yang , N. Zhao , Z. Li , and X. Cao , “Gastrodin Protects Dopaminergic Neurons via Insulin‐Like Pathway in a Parkinson's Disease Model,” BMC Neuroscience 20, no. 1 (2019): 31. 31208386 10.1186/s12868-019-0512-x PMC6580469

[24] D. Wang , “Toxicity Assessment Under the Pathological Conditions,” Exposure Toxicology in Caenorhabditis Elegans (2020): 653–682.

[25] F. Blandini , M. T. Armentero , and E. Martignoni , “The 6‐Hydroxydopamine Model: News From the Past,” Parkinsonism & Related Disorders 14, no. Suppl 2 (2008): S124–S129. 18595767 10.1016/j.parkreldis.2008.04.015

[26] R. Bodhicharla , A. Nagarajan , J. Winter , et al., “Effects of Alpha‐Synuclein Overexpression in Transgenic *Caenorhabditis Elegans* Strains,” CNS & Neurological Disorders Drug Targets 11, no. 8 (2012): 965–975. 23244416 10.2174/1871527311211080005 PMC3744922

[27] Y. A. Wang , L. van Sluijs , Y. Nie , M. G. Sterken , S. C. Harvey , and J. E. Kammenga , “Genetic Variation in Complex Traits in Transgenic Alpha‐Synuclein Strains of *Caenorhabditis Elegans* ,” Genes 11, no. 7 (2020): 778. 32664512 10.3390/genes11070778 PMC7397059

[28] P. R. Angelova , M. L. Choi , A. V. Berezhnov , et al., “Alpha Synuclein Aggregation Drives Ferroptosis: An Interplay of Iron, Calcium and Lipid Peroxidation,” Cell Death and Differentiation 27, no. 10 (2020): 2781–2796. 32341450 10.1038/s41418-020-0542-z PMC7492459

[29] E. J. Martinez‐Finley , S. Chakraborty , S. Caito , S. Fretham , and M. Aschner , “ *C. elegans* and Neurodegeneration in *Caenorhabditis Elegans*: Anatomy, Life Cycles and Biological Functions,” Advances in Medicine and Biology 44 (2012): 1–46. 32346495 PMC7188451

[30] A. Oranth , C. Schultheis , O. Tolstenkov , et al., “Food Sensation Modulates Locomotion by Dopamine and Neuropeptide Signaling in a Distributed Neuronal Network,” Neuron 100, no. 6 (2018): 1414–1428. 30392795 10.1016/j.neuron.2018.10.024

[31] M. Ezcurra , Y. Tanizawa , P. Swoboda , and W. R. Schafer , “Food Sensitizes *C. elegans* Avoidance Behaviours Through Acute Dopamine Signalling,” EMBO Journal 30, no. 6 (2011): 1110–1122. 21304491 10.1038/emboj.2011.22 PMC3061029

[32] R. T. Tsai , C. W. Tsai , S. P. Liu , et al., “Maackiain Ameliorates 6‐Hydroxydopamine and SNCA Pathologies by Modulating the PINK1/Parkin Pathway in Models of Parkinson's Disease in Caenorhabditis Elegans and the SH‐SY5Y Cell Line,” International Journal of Molecular Sciences 21, no. 12 (2020): 4455. 32585871 10.3390/ijms21124455 PMC7352553

[33] M. Maulik , S. Mitra , A. Bult‐Ito , B. E. Taylor , and E. M. Vayndorf , “Behavioural Phenotyping and Pathological Indicators of Parkinson's Disease in *C. elegans* Models,” Frontiers in Genetics 8, no. 77 (2017), 10.3389/fgene.2017.00077. PMC5468440 28659967

[34] Q. X. Chen , L. Zhou , T. Long , et al., “Galangin Exhibits Neuroprotective Effects in 6‐OHDA‐Induced Models of Parkinson's Disease via the Nrf2/Keap1 Pathway,” Pharmaceuticals (Basel) 15, no. 8 (2022): 1014. 36015161 10.3390/ph15081014 PMC9413091

[35] B. D. Li , Z. Y. Bi , J. F. Liu , et al., “Adverse Effects Produced by Different Drugs Used in the Treatment of Parkinson's Disease: A Mixed Treatment Comparison,” CNS Neuroscience & Therapeutics 23, no. 10 (2017): 827–842. 28872217 10.1111/cns.12727 PMC6492757

[36] Z. Rabiei , K. Solati , and H. Amini‐Khoei , “Phytotherapy in Treatment of Parkinson's Disease: A Review,” Pharmaceutical Biology 57, no. 1 (2019): 355–362. 31141426 10.1080/13880209.2019.1618344 PMC6542178

[37] A. Sharma and G. Kaur , “Tinospora Cordifolia as a Potential Neuroregenerative Candidate Against Glutamate Induced Excitotoxicity: An In Vitro Perspective,” BMC Complementary and Alternative Medicine 18, no. 1 (2018): 268. 30285727 10.1186/s12906-018-2330-6 PMC6167833

[38] M. A. Mori , A. M. Delattre , B. Carabelli , et al., “Neuroprotective Effect of Omega‐3 Polyunsaturated Fatty Acids in the 6‐OHDA Model of Parkinson's Disease Is Mediated by a Reduction of Inducible Nitric Oxide Synthase,” Nutritional Neuroscience 21, no. 5 (2018): 341–351. 28221817 10.1080/1028415X.2017.1290928

[39] S. K. M. Chaves , M. I. Afzal , M. T. Islam , et al., “Palmatine Antioxidant and Anti‐Acetylcholinesterase Activities: A Pre‐Clinical Assessment,” Cellular and Molecular Biology (Noisy‐le‐Grand, France) 66, no. 4 (2020): 54–59. 32583771

[40] H. S. Lim , B. C. Moon , J. Lee , G. Choi , and G. Park , “The Insect Molting Hormone 20‐Hydroxyecdysone Protects Dopaminergic Neurons Against MPTP‐Induced Neurotoxicity in a Mouse Model of Parkinson's Disease,” Free Radical Biology & Medicine 159 (2020): 23–36. 32745769 10.1016/j.freeradbiomed.2020.07.010

[41] W. Yang , Y. H. Chen , H. Liu , and H. D. Qu , “Neuroprotective Effects of Piperine on the 1‐Methyl‐4‐Phenyl‐1,2,3,6‐Tetrahydropyridine‐Induced Parkinson's Disease Mouse Model,” International Journal of Molecular Medicine 36, no. 5 (2015): 1369–1376. 26648012 10.3892/ijmm.2015.2356

[42] D. A. Omoboyowa , T. A. Balogun , O. M. Omomule , and O. A. Saibu , “Identification of Terpenoids From *Abrus precatorius* Against Parkinson's Disease Proteins Using in Silico Approach,” Bioinformatics and Biology Insights 15 (2021): 11779322211050757. 34707350 10.1177/11779322211050757 PMC8544761

[43] K. Kamireddy , S. Chinnu , P. S. Priyanka , P. S. Rajini , and P. Giridhar , “Neuroprotective Effect of Decalepis Hamiltonii Aqueous Root Extract and Purified 2‐Hydroxy‐4‐Methoxy Benzaldehyde on 6‐OHDA Induced Neurotoxicity in *Caenorhabditis elegans* ,” Biomedicine & Pharmacotherapy 105 (2018): 997–1005. 30021395 10.1016/j.biopha.2018.06.002

[44] A. Dubey , N. S. Ghosh , N. Agnihotri , et al., “Herbs Derived Bioactive Compounds and Their Potential for the Treatment of Neurological Disorders,” Clinical Schizophrenia & Related Psychoses 16, no. 2 (2022), 10.3371/CSRP.DANG.081922.

[45] R. Aoki , T. Yagami , H. Sasakura , et al., “A Seven‐Transmembrane Receptor That Mediates Avoidance Response to Dihydrocaffeic Acid, a Water‐Soluble Repellent in *Caenorhabditis elegans* ,” Journal of Neuroscience: The Official Journal of the Society for Neuroscience 31, no. 46 (2011): 16603–16610. 22090488 10.1523/JNEUROSCI.4018-11.2011 PMC6633322

[46] T. Sanguanphun , S. Promtang , N. Sornkaew , N. Niamnont , P. Sobhon , and K. Meemon , “Anti‐Parkinson Effects of *Holothuria leucospilota* ‐Derived Palmitic Acid in *Caenorhabditis elegans* Model of Parkinson's Disease,” Marine Drugs 21, no. 3 (2023): 141. 36976190 10.3390/md21030141 PMC10051922

[47] S. Batelli , R. W. Invernizzi , A. Negro , et al., “The Parkinson's Disease‐Related Protein DJ‐1 Protects Dopaminergic Neurons In Vivo and Cultured Cells From Alpha‐Synuclein and 6‐Hydroxydopamine Toxicity,” Neurodegenerative Diseases 15, no. 1 (2015): 13–23. 25500798 10.1159/000367993

[48] P. Shrivastava , K. Vaibhav , R. Tabassum , et al., “Anti‐Apoptotic and Anti‐Inflammatory Effect of Piperine on 6‐OHDA Induced Parkinson's Rat Model,” Journal of Nutritional Biochemistry 24, no. 4 (2013): 680–687. 22819561 10.1016/j.jnutbio.2012.03.018

[49] K. Hu , X. Chen , W. Chen , et al., “Neuroprotective Effect of Gold Nanoparticles Composites in Parkinson's Disease Model,” Nanomedicine: Nanotechnology, Biology and Medicine 14, no. 4 (2018): 1123–1136. 29474924 10.1016/j.nano.2018.01.020

[50] G. Gao , R. Chen , M. He , et al., “Gold Nanoclusters for Parkinson's Disease Treatment,” Biomaterials 194 (2019): 36–46. 30576972 10.1016/j.biomaterials.2018.12.013

[51] H. Xicoy , B. Wieringa , and G. J. M. Martens , “The Role of Lipids in Parkinson's Disease,” Cells 8, no. 1 (2019): 27. 30621069 10.3390/cells8010027 PMC6356353

[52] P. A. Postila and T. Róg , “A Perspective: Active Role of Lipids in Neurotransmitter Dynamics,” Molecular Neurobiology 57, no. 2 (2020): 910–925. 31595461 10.1007/s12035-019-01775-7 PMC7031182

[53] P. Godkar , R. K. Gordon , A. Ravindran , and B. P. Doctor , “Celastrus Paniculatus Seed Water Soluble Extracts Protect Cultured Rat Forebrain Neuronal Cells From Hydrogen Peroxide‐Induced Oxidative Injury,” Fitoterapia 74, no. 7–8 (2003): 658–669. 14630170 10.1016/s0367-326x(03)00190-4

[54] J. Anjaneyulu , V. R , and A. Godbole , “Differential Effect of Ayurvedic Nootropics on *C. elegans* Models of Parkinson's Disease,” Journal of Ayurveda and Integrative Medicine 11, no. 4 (2020): 440–447, 10.1016/j.jaim.2020.07.006. 32978047 PMC7772502

[55] E. Deepak , D. Swati , and D. S. Dhruw , “Effect of Rajat Bhasma With Smritisagar Rasa in Parkinson,” Journal of Ayurveda and Integrated Medical Sciences 2, no. 4 (2017): 146–150.

[56] J. Liu , W. Liu , R. Li , and H. Yang , “Mitophagy in Parkinson's Disease: From Pathogenesis to Treatment,” Cells 8, no. 7 (2019): 712. 31336937 10.3390/cells8070712 PMC6678174

[57] H. Aspernig , T. Heimbucher , W. Qi , et al., “Mitochondrial Perturbations Couple mTORC2 to Autophagy in *C. elegans* ,” Cell Reports 29, no. 6 (2019): 1399–1409. 31693882 10.1016/j.celrep.2019.09.072

[58] B. Xiao , J. Kuruvilla , and E. K. Tan , “Mitophagy and Reactive Oxygen Species Interplay in Parkinson's Disease,” NPJ Parkinsons Disease 8, no. 1 (2022): 135. 10.1038/s41531-022-00402-y PMC9579202 36257956

[59] M. Borsche , S. L. Pereira , C. Klein , and A. Grunewald , “Mitochondria and Parkinson's Disease: Clinical, Molecular, and Translational Aspects,” Journal of Parkinson's Disease 11, no. 1 (2021): 45–60. 10.3233/JPD-201981 PMC7990451 33074190

[60] B. Li , D. An , and S. Zhu , “PBX1 Attenuates 6‐OHDA‐Induced Oxidative Stress and Apoptosis and Affects PINK1/PARKIN Expression in Dopaminergic Neurons via FOXA1,” Cytotechnology 74, no. 2 (2022): 217–229. 35464170 10.1007/s10616-021-00518-8 PMC8975925

[61] D. Mundekkad and W. C. Cho , “Mitophagy Induced by Metal Nanoparticles for Cancer Treatment,” Pharmaceutics 14, no. 11 (2022): 2275. 36365094 10.3390/pharmaceutics14112275 PMC9699542

[62] N. G. Kim , D. J. Jung , Y. K. Jung , and K. S. Kang , “The Effect of a Novel Mica Nanoparticle, STB‐MP, on an Alzheimer's Disease Patient‐Induced PSC‐Derived Cortical Brain Organoid Model,” Nanomaterials (Basel) 13, no. 5 (2023): 893. 36903771 10.3390/nano13050893 PMC10005775

[63] M. Xia , D. Huang , Y. Tong , and J. Lin , “Pearl Powder Reduces Sleep Disturbance Stress Response Through Regulating Proteomics in a Rat Model of Sleep Deprivation,” Journal of Cellular and Molecular Medicine 24, no. 9 (2020): 4956–4966. 32220128 10.1111/jcmm.15095 PMC7205811

[64] H. L. Yang , M. Korivi , M. K. Lin , et al., “Antihemolytic and Antioxidant Properties of Pearl Powder Against 2,2′‐Azobis(2‐Amidinopropane) Dihydrochloride‐Induced Hemolysis and Oxidative Damage to Erythrocyte Membrane Lipids and Proteins,” Journal of Food and Drug Analysis 25, no. 4 (2017): 898–907. 28987367 10.1016/j.jfda.2016.10.007 PMC9328879

[65] J. T. BK , I. Arnoldussen , C. Vriend , and O. van de Rest , “Dietary Approaches to Improve Efficacy and Control Side Effects of Levodopa Therapy in Parkinson's Disease: A Systematic Review,” Journal Advances in Nutrition 12, no. 6 (2021): 2265–2287, 10.1093/advances/nmab060. 34113965 PMC8634393

